# A “human knockout” model to investigate the influence of the α-actinin-3 protein on exercise-induced mitochondrial adaptations

**DOI:** 10.1038/s41598-019-49042-y

**Published:** 2019-09-03

**Authors:** I. D. Papadimitriou, N. Eynon, X. Yan, F. Munson, M. Jacques, J. Kuang, S. Voisin, K. N. North, D. J. Bishop

**Affiliations:** 1 Institute for Health and Sport (iHeS), Victoria University, Melbourne, Australia; 2 Murdoch Children’s Research Institute, Melbourne, Australia; 3 Department of Physiology, Mahidol University, Bangkok, Thailand; 4 School of Medical & Health Sciences, Edith Cowan University, Joondalup, Australia

**Keywords:** Molecular biology, Physiology

## Abstract

Research in α-actinin-3 knockout mice suggests a novel role for α-actinin-3 as a mediator of cell signalling. We took advantage of naturally-occurring human “knockouts” (lacking α-actinin-3 protein) to investigate the consequences of α-actinin-3 deficiency on exercise-induced changes in mitochondrial-related genes and proteins, as well as endurance training adaptations. At baseline, we observed a compensatory increase of α-actinin-2 protein in ACTN3 XX (α-actinin-3 deficient; n = 18) vs ACTN3 RR (expressing α-actinin-3; n = 19) participants but no differences between genotypes for markers of aerobic fitness or mitochondrial content and function. There was a main effect of genotype, without an interaction, for RCAN1-4 protein content (a marker of calcineurin activity). However, there was no effect of genotype on exercise-induced expression of genes associated with mitochondrial biogenesis, nor post-training physiological changes. In contrast to results in mice, loss of α-actinin-3 is not associated with higher baseline endurance-related phenotypes, or greater adaptations to endurance exercise training in humans.

## Introduction

An important component of the skeletal muscle Z-disk in fast-twitch muscle fibres is α-actinin-3^1^, a protein that interacts with multiple metabolic, structural, and signalling molecules^2^. A common polymorphism in the *ACTN3* gene (i.e., the *ACTN3* 577XX genotype) leads to complete absence of α-actinin-3 protein in the fast-twitch fibres of skeletal muscles. This genotype is underrepresented in elite Australian^3^, Finish^4^, Greek^5^, Russian^6^, Israeli^7^, Polish^8^, and Japanese^9^, power-oriented athletes, which suggests α-actinin-3 deficiency has a negative effect on the function of fast-twitch muscle fibres^10^. There are, however, contradicting findings concerning the influence of the *ACTN3* 577XX genotype on endurance performance. Although some studies have reported an association between the *ACTN3* 577XX genotype and endurance status in elite athletes^11^, others have not^12,13^.

In contrast to human studies, research in *Actn3* knockout mice (*Actn3* KO) suggests that the adaptive response to endurance training is influenced by the *ACTN3* genotype^14^. After four weeks of endurance training, *Actn3* KO mice had greater endurance exercise performance and faster recovery from fatigue, compared with wild type (WT) mice, and this was associated with a shift in the characteristics of fast-twitch muscle fibres toward a more oxidative, slow-twitch phenotype^15,16^. Seto *et al*.^14^ also reported an increase in calcineurin activity (1.9-fold) in the muscle of exercised *Actn3* KO mice compared with the WT mice (p = 0.093), which was associated with an increase (2.9-fold) in the Regulator of Calcineurin (RCAN1-4) protein content^17^. Consistent with their observations in mice, there was a also greater protein content of RCAN1-4 in resting muscle samples obtained from *ACTN3* 577XX versus *ACTN3* 577RR humans^14^.

The molecular mechanisms underlying the modified calcineurin activity when α-actinin-3 is absent appear to be via altered binding of calsarcin-2 to sarcomeric α-actinins. When the α-actinin-3 protein is absent (*ACTN3* 577XX) there is a compensatory increase in α-actinin-2, which binds more tightly to calsarcin-2 (a negative regulator of calcineurin)^18^. Thus, absence of α-actinin-3 protein (with a compensatory increase in α-actinin-2) has been hypothesised to increase the release and activation of calcineurin^14^. Activated calcineurin is able to dephosphorylate many substrates, including Nuclear Factor of Activated T-cells (NFAT) - promoting translocation of this protein to the nucleus^19^. In the nucleus, NFAT interacts with myocyte enhancer factor-2 (MEF2) to regulate expression of peroxisome proliferator-activated receptor gamma coactivator 1 alpha (PGC-1α) – often described as the “master regulator” of mitochondrial biogenesis^20^. Compared to WT mice, overexpression of activated calcineurin results in a significant increase in electron transport system proteins (complexes I to V)^21^, and a 35% increase in resting mitochondrial respiratory capacity^22^, and a significant increase in PGC-1α protein content^23^. Altered calcineurin activity therefore provides a plausible biological mechanism for the more “aerobic” or “endurance” phenotype in mice that lack α-actinin-3.

Knocking out the *ACTN3* gene in mice has provided a useful model to investigate the effects of α-actinin-3 on skeletal muscle metabolism and associated molecular signalling pathways. Nonetheless, results from KO mice don’t always translate to humans and complementary studies in humans remain essential to confirm the findings observed in mice. While knocking out genes in humans is ethically off-limits, the high percentage of the human population in which the α-actinin-3 protein is absent (i.e., naturally occurring human “knockouts”) affords us the unique opportunity to study the consequences of α-actinin-3 deficiency on human skeletal muscle.

Our aim was to investigate the role of the α-actinin-3 protein in regulating metabolism, molecular signalling pathways, and physiological adaptations, in human skeletal muscle at baseline, following one session of High-Intensity Interval Exercise (HIIE), and following 4 weeks of High-Intensity Interval Training (HIIT). We chose HIIT as it has been reported to be equally or more effective at improving mitochondrial respiratory function, and increasing the expression of nuclear genes encoding mitochondrial proteins (NUGEMPs), compared to moderate-intensity exercise^24–26^. We hypothesised that humans with the *ACTN3* XX genotype (i.e., *Actn3* human “knockouts”) would have greater endurance performance and higher mitochondrial content and respiratory function at baseline compared to their *ACTN3* RR counterparts (α-actinin-3 protein is present). We also hypothesised that *ACTN3* XX humans would have higher mitochondrial-related gene and protein expression following a single session of HIIE, and greater changes in physiological characteristics important for endurance performance following four weeks of HIIT, compared to their *ACTN3* RR counterparts.

## Methods

### Study design and participants

More than 100 participants were recruited from the local community. Informed consent was obtained prior to a subject’s participation in the research project. The experimental protocols/methods used in this study were approved by the Victoria University Ethics Committee (Ethics Application ID: HRE13-223) and carried out based on our standardised protocols^27^ in accordance with Victoria University relevant guidelines, research ethics, and regulations regarding the use of human tissue and DNA samples.

After screening for *ACTN3* genotype, only the *ACTN3* RR and the *ACTN3* XX participants were included in this study to ensure we compared the two homozygotic genotypes (i.e., RR vs. XX). Only healthy Caucasians (for ≥3 generations), with a Body Mass Index (BMI) between 20 and 30 kg•m^−2^ and a body fat value < 25%, were included in the study to ensure both body composition and genetic homogeneity^28^. Participants with a history of any of the following medical conditions were excluded from the study: a significant recurrent or chronic respiratory condition, possible coronary heart disease, major musculoskeletal problems or conditions interfering with the ability to cycle, uncontrolled metabolic disorders or endocrine disorders, or diabetes requiring insulin.

The calculation of sample size was based on a conservative estimate of both the expected change and the standard deviation for exercise-induced changes in PGC-1α mRNA (derived from our previous work). The sample size necessary to achieve statistical significance between groups (using an alpha of 0.05 and a power of at least 0.80) was 15 participants for each group. To account for the possibility of drop outs, we aimed to recruit at least 18 participants for each group.

Thirty-seven (n = 19 *ACTN3* RR, and n = 18 *ACTN3* XX) moderately-trained (VO_2_ peak 35 to 60 mL•min^−1^•kg^−1^) men, with a BMI of 26.6 ± 2.6 for RR and a BMI of 24.2 ± 2.4 for XX, with a mean age 32.2 ± 7.1 years for RR and 29.4 ± 8.4 years for XX, were subsequently included in our study population.

### Genotyping

Genomic DNA was extracted from fingertip blood samples obtained from the graded exercise tests (GXT) using the GeneJET Genomic Whole Blood DNA Purification Kit (#K0781 Thermo Scientific, MA, USA). *ACTN3* gene variants were determined in duplicate using the TaqMan SNP assay (Applied Biosystems, Thermo Fisher Scientific, CA, USA) by Mastercycler® ep realplex2 (Eppendorf, Hamburg, Germany). Genotyping was replicated in another independent institute (The Murdoch Childrens Research Institute, Melbourne), as previously described^29^, to validate the results.

### Pre-training physical activity monitoring

To determine if there were any fluctuations in habitual physical activity between participants, 1 week prior to commencing the study the participants’ activity levels were monitored for one week. The monitoring of each participant’s activity level was performed using an ActiGraph GT3X + device (ActiGraph LLC, Fort Walton Beach, FL, USA). The GT3X + activity monitor has been used in many types of and clinical applications^30^ and research^31^. The activity monitor includes a micro-electro-mechanical system based tri-axis accelerometer sensor that provides measures of acceleration in three axes, with a composite measure called the vector magnitude (VM = √(x2 + y2 + z2)). The accelerometer has a ±6 g range, with a sampling rate ranging from 30 Hz to 100 Hz (user selectable), and stores the raw, non-filtered/accumulated data in the units of gravity. The activity monitor can sample continuously for between 24 and 32 days depending on the selected sampling frequency.

The ActiGraph GT3X+ device was worn either over or under clothing, which ever was most comfortable for the participant and threaded onto an elastic belt. The device was positioned snugly enough against the body so that it could not flip around. The accelerometer was worn with the elastic belt fastened around the waist over the right hip bone all day while the participant was awake. The device was removed only when the participant was in bed at night, showering, or swimming. A daily diary assisted in monitoring when the device was removed or if any water-based activities were performed. The acceleration data were downloaded from the activity monitor and processed over a user-specified time sampling interval. Activity intensity and energy expenditure was calculated using algorithms that have been used in other similar studies^32^.

### Nutrition consultation

To ensure similar nutrition between participants, each participant was provided with individualised, pre-packaged meals for the 48 hours prior to all resting muscle biopsies and standardised nutrition (composed of mainly liquids and carbohydrates) before and after each training session during the 4-week training period. The energy content of the provided meals was calculated using the participant’s body mass, the Mifflin-St-Jeor equation, and the participant’s, age, and height^33^. The Foodworks (Xyris) nutritional database was used to determine the nutritional components of the packaged meals and to ensure all nutritional requirements were met with the diet. The content of the diets was constructed based on the current Medical Research Council (NHMRC) and National Health guidelines. To ensure adequate access to carbohydrate energy stores, participants were asked to consume a pre-packaged training meal of high glucose food items (1 to 1.5 g•kg^−1^ BM) 2 hours prior to the commencement of each testing session, according to Australian Institute of Sport (AIS) guidelines (https://www.ausport.gov.au/ais/sports_nutrition), composed of mainly liquids and carbohydrates. Participants were also asked to refrain from caffeine and alcohol during the dietary control period, which was 48 hours prior to all biopsies.

### Performance tests

Prior to the start of the exercise intervention phase, all participants completed familiarisation sessions and baseline testing. All visits were separated by a minimum of 48 hours. In addition, participants were required to refrain from alcohol, caffeine consumption, and any form of physical activity, for 24 hours before all performance tests. The familiarisation and baseline testing consisted of the following^27^:

1. 20-km cycle Time Trial (20k-TT) – The 20k-TT test was performed on a Velotron® stationary cycle ergometer (RacerMate Inc. Seattle, WA, USA). Participants completed a warm-up consisting of 5 minutes of cycling at 60 W. Following a 2-minute rest, participants were then required to complete the 20-k TT in the quickest possible time. Participants were permitted to monitor their progress through completed distance and were provided with verbal encouragement during the test.
2. Graded exercise test to exhaustion (GXT) – The GXT was performed on an electronically-braked cycle ergometer (Lode-Excalibur sport, Groningen, the Netherlands) and consisted of 4-min stages of increasing intensity, separated by 30-s rest periods, until exhaustion^34^. The test started at 60, 90, or 120 W (depending on the participant’s 20k-TT results) and increased by 30 W in each subsequent stage. For the participants who did not complete the final stage, peak power (W_peak_) was calculated based on power achieved at the point of exhaustion. Fingertip samples were taken at rest, after each completed stage, and immediately following exhaustion, and were analysed by a YSI 2300 STAT Plus system (Yellow Springs, Ohio, USA). The LT was then calculated using the modified D_max_ method, which is determined by the point on the polynomial regression curve that yields the maximum perpendicular distance to the straight line connecting the first increase in lactate concentration above the resting value and the final lactate point^35^. The average of the two baseline GXT tests was recorded as the baseline value and was also used to customise subsequent exercise intensities if the difference was no more than 5%; otherwise, the highest value was used. Residual capillary blood samples were saved for DNA analysis^27^.
3. VO_2peak_ test - After five minutes of rest following each GXT, peak oxygen consumption (VO_2peak_) was measured using a calibrated Quark CPET metabolic system (COSMED, Rome, Italy). Briefly, participants wore the Cosmed face mask and exercised to exhaustion at 105% of the W_peak_ measured during the previous GXT. The VO_2peak_ was considered the highest value in 1 minute obtained during the test, and the average of the two baseline tests was recorded as the baseline value. Previous studies have reported the VO_2peak_ measured this way is not different from that derived from a ramp test^36^.

### Muscle biopsies and high-intensity interval exercise (HIIE)

Forty-eight to 72 hours after the last VO_2peak_ test, muscle biopsies were collected from the vastus lateralis muscle of the participant’s dominant leg. Following injection of a local anaesthetic (5 mL, 1% Xylocaine), incisions were made and the biopsy needle was inserted. Muscle samples were collected with manual suction applied^37^. The samples (50 to 200 mg) were then immediately blotted on filter paper to remove excess blood, and a small portion (5 to 15 mg) was immediately processed for the determination of mitochondrial respiration *in SITU*^38^; the remaining muscle was snap frozen in liquid nitrogen before being stored at −80 °C for further analyses. Following the pre-training baseline muscle biopsy, participants performed a single session of HIIE on an electronically-braked cycle ergometer (Velotron®, Racer Mate Inc, Seattle, USA). The session consisted of eight 2-minute intervals performed at an intensity between the individually-determined pre-training LT power and W_peak_ (LT + 40% × [W_peak_ − LT)), which were interspersed with 1-minute recovery periods at 60 W (work-to-rest ratio of 2:1). All participants were motivated and reached the expected loads during all eight 2-minute intervals. Muscle biopsy samples were also taken immediately after and 3 hours post the HIIE session^27^.

### High intensity interval training (HIIT) phase

**Figure 1:**
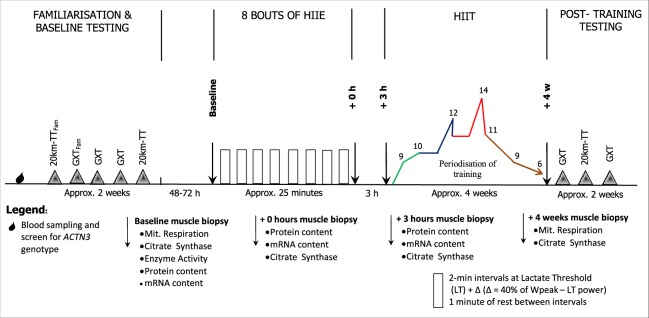
Study overview. 20k-TTfam: 20 kilometre time-trial familiarisation; 20k-TT: 20 kilometre time trial; GXTfam: graded exercise test familiarisation; GXT: graded exercise test; LT: Power at the Lactate Threshold, Wpeak: peak power achieved during the GXT, Δ: difference between Wpeak and LT; The periodisation of training ranged from 9 bouts of HIIE at LT + 40%Δ in the first week (green line), to one peak of 12 bouts at LT + 50%Δ in the second week (blue line), and one higher peak of 14 bouts at LT + 60%Δ in the third week. The intensity increased to LT + 70%Δ in the fourth week, with a reduction in the number of bouts to 6 at the end of this last week (brown line) - this served as taper period. mRNA: messenger RNA.

Two days after the collection of muscle biopsies, participants were required to train 3 times per week for 4 weeks (12 sessions including the HIIE session with biopsies) (Fig. 1). All training sessions where completed on an electronically-braked cycle ergometer (Velotron®, Racer Mate Inc., Seattle, USA). Each session consisted of six to fourteen 2-min intervals performed at intensities ranging from LT + 40% × [W_peak_ − LT] (week 1) to LT + 70% × [W_peak_ − LT] (week 4), and interspersed with 1-min recovery periods at 60 W (work- to-rest ratio of 2:1). In order to maintain progression, there were a different numbers of bouts per session (as shown in Fig. 1).

### Post-training performance tests

At the completion of training, post-training GXTs and VO_2peak_ tests took place at the same time of day as pre-training to assess changes in W_peak_ and LT (Fig. 1). The average of the two GXT tests was used to determine the influence of training on common physiological determinants of endurance performance if the difference was less than 5%. If the difference between the results of the two tests was larger than 5%, the highest value was used. During a second visit the participants performed a post-training 20K-TT test to assess improvements in endurance performance (Fig. 1)^27^.

### RNA extraction and gene expression analysis

Total RNA was extracted from approximately 15 mg of frozen muscle. Cellular membranes were dissociated in TRIzol® Reagent (Invitrogen, Melbourne, Australia) through TissueLyser II (Qiagen, Hilden, Germany) for 2 × 1 minute at 30 Hz. The homogenate was centrifuged (13,000 RPM for 15 minutes) and the RNA containing supernatant removed. The homogenate then was combined with chloroform (Sigma-Aldrich, St Louis, USA) and total tissue RNA was then extracted using the TRIzol protocol according to the manufacturer’s instructions, with the exception of RNA precipitation which was conducted for a minimum of 2 hours at −20 °C in the presence of 10 µL of 5 M sodium chloride. RNA purity was checked using the ratio of its absorbance at 260 and 280 nm using a BioSpectrometer (Eppendorf, Hamburg, Germany) and concentration was quantified spectrophotometrically at 260 nm. First strand cDNA was then generated from 1 µg of template RNA using the commercially available iScript™ cDNA synthesis kit (Bio-Rad Laboratories, Hercules, USA) using random hexamers and oligo dTs according to the protocol provided with the iScipt cDNA synthesis kit (Bio-Rad Laboratories, Hercules, USA). cDNA was stored at −20 °C for subsequent analysis. All samples and reverse transcriptase (RT) negative controls were run together to prevent technical variation. Forward and reverse primers for the target and housekeeping genes were designed based on NCBI RefSeq using NCBI Primer-BLAST (www.ncbi.nlm.nih.gov/BLAST/) (Table 1). Specificity of the amplified product was confirmed by melting point dissociation curves generated by the PCR instrument. The mRNA expression of housekeeping and target genes were quantified by quantitative real-time RT-PCR (QuantStudioTM 7 Flex Real-Time PCR System (Life Technologies, Thermo Fisher Scientific, Wilmington, DE, USA), using a 5 µL PCR reaction volume and SYBR® Green chemistry (iTaqTM Universal SYBR® Green Supermix, Bio-Rad, Hercules, CA). All samples were run in duplicate simultaneously with template free controls, using an automated pipetting system (epMotion 5073, Eppendorf, Hamburg, Germany). The following PCR cycling patterns were used: initial denaturation at 95 °C for 3 min, 40 cycles of 95 °C for 15 s and 60 °C for 60 s.

**Table 1:** Details of PCR primers used for RT-qPCR.

Gene	Forward Sequence	Reverse Sequence
*PGC-1α*	5′-CAGCCTCTTTGCCCAGATCTT-3′	5′-TCACTGCACCACTTGAGTCCAC-3′
*PGC-1α1*	5′-ATGGAGTGACATCGAGTGTGCT-3′	5′-GAGTCCACCCAGAAAGCTGT-3′
*PGC-1α4*	5′-TCACACCAAACCCACAGAGA-3′	5′-TCACACCAAACCCACAGAGA-3′
*COX4-1*	5′-GAGCAATTTCCACCTCTGC-3′	5′-CAGGAGGCCTTCTCCTTCTC-3′
*NDUFB3*	5′-TCAGATTGCTGTCAGACATGG-3′	5′-TGGTGTCCCTTCTATCTTCCA-3′
*SDHB*	5′-AAATGTGGCCCCATGGTATTG-3′	5′-AGAGCCACAGATGCCTTCTCTG-3′
*PDK4*	5′-GCAGCTACTGGACTTTGGTT-3′	5′-GCGAGTCTCACAGGCAATTC-3′
*MFN2*	5′-CCCCCTTGTCTTTATGCTGATGTT-3′	5′-TTTTGGGAGAGGTGTTGCTTATTTC-3′
*RCAN1-4*	5′-GGGTCTGTAGCGCTTTCACT-3′	5′-GGACTCAAATTTGGCCCTGG-3′
*SOD-1*	5′-GGTCCTCACTTTAATCCTCTAT-3′	5′-CATCTTTGTCAGCAGTCACATT-3′
*HSP70*	5′-GGGCCTTTCCAAGATTGCTG-3′	5′-TGCAAACACAGGAAATTGAGAACT-3′
*VEGF*	5′-ACAACAAATGTGAATGCAGACCAA-3′	5′-CGTTTTTGCCCCTTTCCCTT-3′
*GAPDH*	5′-AATCCCATCACCATCTTCCA-3′	5′-TGGACTCCACGACGTACTCA-3′
*B2M*	5′-TGCTGTCTCCATGTTTGATGTATCT-3′	5′-TCTCTGCTCCCCACCTCTAAGT-3′
*TBP*	5′-CAGTGACCCAGCAGCATCACT-3′	5′-AGGCCAAGCCCTGAGCGTAA-3′
*Cyclophilin*	5′-GTCAACCCCACCGTGTTCTTC-3′	5′-TTTCTGCTGTCTTTGGGACCTTG-3′
*18S*	5′-CTTAGAGGGACAAGTGGCG-3′	5′-GGACATCTAAGGGCATCACA-3′
*ACTB*	5′-GAGCACAGAGCCTCGCCTTT-3′	5′-TCATCATCCATGGTGAGCTGGC-3′

PGC-1α, peroxisome proliferator-activated receptor-ɣ coactivator 1α; COX4-1, cytochrome c oxidase subunit 4I1; NDUFB3, NADH:ubiquinone oxidoreductase subunit B3; SDHB, succinate dehydrogenase complex iron sulfur subunit B; PDK4, pyruvate dehydrogenase kinase 4; MFN2, mitofusin 2; RCAN1-4, regulator of calcineurin 1-4; SOD-1, superoxide dismutase 1; HSP-70, heat shock protein family A (Hsp70) member 4; VEGF, vascular endothelial growth factor A; GAPDH, glyceraldehyde 3-phosphate dehydrogenase; B2M, β-2-microglobulin; TBP, TATA-box binding protein; Cyclophilin, peptidylprolyl isomerase A; 18S, 18S ribosomal RNA; ACTB, actin beta.

### Western blot for protein content

Approximately 10 mg of frozen muscle samples were homogenised in ice-cold RadioImmunoPrecipitation Assay (RIPA) lysis buffer (50 mM Tris-HCl, pH 7.4, 150 mM NaCl, 0.5% Sodium Deoxycholate, 1% Triton X-100, 0.1% SDS, 1 mM EDTA with protease/phosphatase inhibitors, 1 mM PMSF, 1 g/mL Aprotinin, 1 g/ml Leupeptin, 1 mM Benzamidine, 1 mM Na3VO4, 5 mM Na Pyrophosphate, 1 mM DTT and 1 mM NaF) using a TissueLyser II (Qiagen, Hilden, Germany) for 2 × 1 minute at 30 Hz, and rotated for 1 h at 4 °C. Muscle lysates were stored at −80 °C until further analysis. Total protein content of muscle lysates was determined using the bicinchoninic acid assay.

Protein extracts were loaded on sodium dodecyl sulfate-polyacrylamide gels, separated for 120 minutes at 100 V and subsequently transferred to PolyVinyl DiFluoride (PVDF) membranes (Bio-Rad Laboratories, Hercules, USA) using a Bio-Rad blot system for 100 minutes at 100 V. Thereafter, blots were blocked for 60 minutes in 5% milk in tris-buffered saline (TBS) and washed with TBS plus 0.1% Tween at room temperature, followed by incubation with different primary antibodies overnight at 4 °C. After washing, the membranes were incubated with the appropriate secondary antibodies for 60 minutes at room temperature and revealed using a chemiluminescent substrate (Bio-Rad Laboratories, Hercules, USA). The primary antibodies were ACTN3 (a kind gift from Professor Kathryn North), ACTN2 (Ab68168, Abcam, Cambridge, United Kingdom), PGC-1α (#2178, Cell Signaling, Danvers, Massachusetts, United States), RCAN1 (D6694, Sigma-Aldrich, St. Louis, Missouri, United States), Calsarcin 2 (Ab197660, Abcam). All primary antibodies were diluted 1:1000 in 5% BSA. The secondary antibodies were purchased from Perkin Elmer (Waltham, Massachusetts, USA) and diluted 1:10000 in 5% skim milk (Goat Anti-Mouse IgG (NEF822001EA), Goat Anti-Rabbit IgG (NEF812001EA)). Light emission was recorded using ChemiDocTM MP System (Bio-Rad Laboratories, Hercules, USA) and quantified by image analysis software (Image Lab, Bio-Rad). Protein content was then normalised to totally protein analysis by TGX stain-free gel (Bio-Rad Laboratories, Hercules, USA)^39^.

### Mitochondrial respiration and citrate synthase

Immediately after the resting biopsy, muscle fibres were separated gently on ice under a binocular microscope in BIOPS solution (2.77 mM CaK_2_EGTA, 7.23 mM K_2_EGTA, 5.77 mM Na_2_ATP, 6.56 mM MgCl_2_6•H_2_O, 20 mM Taurine, 15 mM Na_2_Phosphocreatine, 20 mM Imidazole, 0.5 mM Dithiothreitol, and 50 mM MES at PH7.1), and permeabilised in the same solution with 50 μg/mL of saponin (Sigma-Aldrich, St Louis, USA) for 30 minutes. This was followed by rinsing the muscle fibres for 3 × 7 minutes in mitochondrial respiration medium on ice (0.5 mM EGTA, 3 mM MgCl_2_•6H_2_O, 60 mM K-lactobionat, 20 mM Taurine, 10 mM KH_2_PO_4_, 20 mM Hepes, 110 mM sucrose, and 1 g•L^−1^ bovine serum albumin at pH 7.1). Experiments were performed on washed muscle fibres under continuous stirring using an oxygraph-2k respirometer (Oroboros Instruments, Austria), containing 2 mL of mitochondrial respiration medium with additional substrates at 37 °C. The following substrates were added (final concentration): malate (2 mM) and pyruvate (5 mM) to support electron entry to complex I (CI); MgCl_2_ (3 mM) and ADP (5 mM) to measure Oxidative phosphorylation (OXPHOS) capacity; Succinate (10 mM) to stimulate CI + II-linked respiration and provide convergent electron input into the Q-junction simultaneously (CI + IIP)^40^. A maximal respiratory capacity is reached when these substrates are present in the respirometer chamber. Cytochrome c (10 µM) was used to test the integrity of the outer mitochondrial membrane^31^. Electron transfer system capacity (ETS with CI + II-linked substrates, CI + IIE) was tested by titrating p-trifluoromethoxy phenylhydrazone (FCCP) (steps of 0.5 µM) until maximal noncoupled respiration was reached. Rotenone (0.5 µM) was then added to block the activity of complex so that electrons could only enter through complex II (CII). Antimycin (3.75 µM) was added to block the activity of complex III and to measure the non-mitochondrial respiration. Different ratios (substrate and coupling control ratios) were calculated from the different titration steps obtained from the protocol used.

CS activity was determined spectrophotometrically at 30 °C by measuring the appearance of the CoA-SH^38^.The reaction was carried out in 100 mM Tris buffer buffer with 0.48 mM acetyl CoA and 0.1 mM 5,59-dithiobis (2-nitrobenzoic acid) (pH 8.3). CS activity was determined in triplicate on a microtiter plate by adding muscle homogenate. After addition of oxaloacetic acid to final concentration of 0.6 mM, the plate was immediately placed in an xMark-Microplate spectrophotometer (Bio-Rad) at 30 °C, and after 30 s of linear agitation, absorbance at 412 nm was recorded every 15 s for 3 min. CS activity were expressed as moles per hour per kilogram protein.

### Statistical analysis

The quantitative genetic association data, mRNA content, protein relative abundance, and maximal mitochondrial respiration data were analysed using linear mixed models, with time point (baseline, post, 3 h post or pre- and post-training) and genotype (RR or XX) as covariates. The interaction between time and genotype was also tested. To test the effect of each covariate (time, genotype or time:genotype), a full model containing all covariates was compared with a null model missing the covariate of interest, using a likelihood ratio test. All data analyses were conducted with the R statistical software with the lme4 and lrtest packages. The significance for all tests was set at 5% (p-value < 0.05).

## Results

### Genotyping

**Figure 2:**
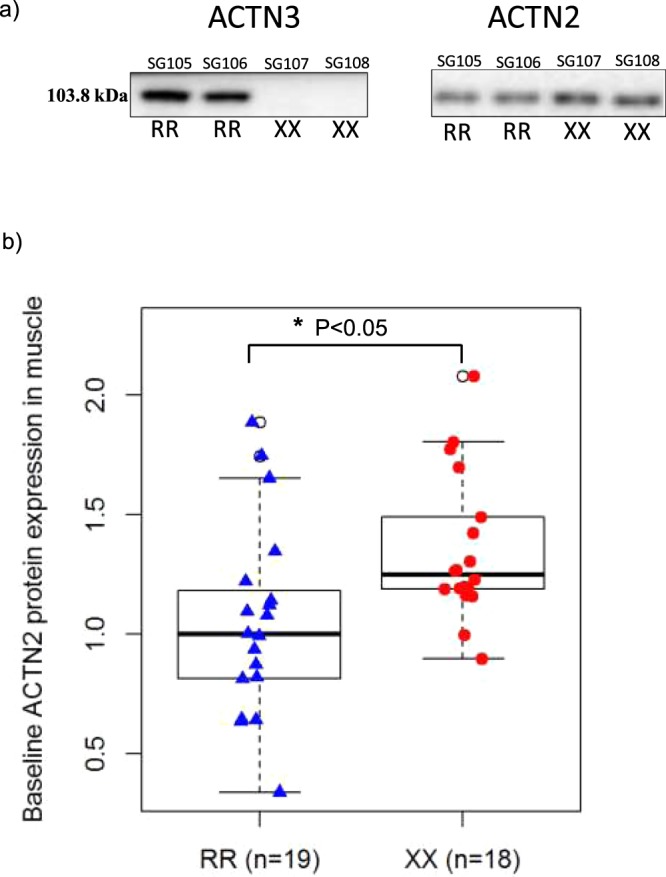
(**a**) Representative images of α-actinin-2 and α-actinin-3 protein content in ACTN3 RR vs. ACTN3 XX participants at baseline. α-actinin-3 protein was present in ACTN3 RR and not in ACTN3 XX participants. (**b**) Box blot displaying α-actinin-2 protein content in ACTN3 RR vs. XX participants at baseline. There was a compensatory greater ACTN2 protein content in ACTN3 XX vs ACTN3 RR participants (p = 0.018). The centre rectangle includes the mean and the interquartile range (IQR). The “whiskers” above and below the box show the locations of the maximum and minimum values (excluding outliers – defined as ≥0.15 × IQR above the third quartile or below the first quartile and indicated by an unfilled circle). *Significant difference between genotypes (p < 0.05).

Genotyping was performed in duplicate and verified by blotting for the α-actinin-3 protein (Fig. 2a). As shown in the representative blots, the α-actinin-3 protein was present in *ACTN3* RR and not *ACTN3* XX participants. There was also a compensatory greater α-actinin-2 protein content in *ACTN3* XX vs *ACTN3* RR participants (p = 0.018), as shown in the representative images (Fig. 2a) and summarised in Fig. 2b.

### Baseline habitual physical activity

There were no significant differences for habitual physical activity measured for seven consecutive days, 1 week prior to commencing the study, between *ACTN3 XX* and *ACTN3 RR* participants (790.2 ± 382.5 Kcal per day for *ACTN3* RR vs. 673.9 ± 343.2 Kcal per day for *ACTN3* XX genotypes; p = 0.37).

### Baseline physiological characteristics

There were no significant differences for the LT, VO_2peak_, W_peak_, 20-k TT performance, citrate synthase activity, or maximal mitochondrial respiration, between *ACTN3 XX* and *ACTN3 RR* participants at baseline (Table 2).

**Table 2:** Endurance-related phenotypes measured at baseline: Lactate threshold, VO_2peak_, W_peak_, 20-km Time-trial performance, Mitochondrial respiration, and Citrate synthase activity for the two ACTN3 genotypes (n = 37).

Phenotype	Genotype	
	RR (n = 19)	XX (n = 18)
Lactate Threshold (W)	210.4 (52)	196.7 (65.9)
VO_2 peak_ (mL•min^−1^•kg^−1^)	43.3 (6.2)	46.9 (6.6)
Lactate Threshold as a percent of Power Peak (%)	71.6 (5.7)	67.8 (7.4)
Peak Power (W_peak_)	291.4 (57.6)	272.1 (70.3)
Time-Trial Performance (s)	2246 (311)	2377.5 (301)
Mitochondrial Respiration (pmol O_2_/s^−1^·mg wet weight^−1^)	111.1 (35.7)	112.3. (37.3)
Citrate synthase activity (mol·h^−1^ · kg protein^−1^)	13.9 (3.3)	13.0 (4.0)

Values are reported as Mean (SD). No significant difference was demonstrated between RR and XX genotypes for any of these phenotypes (p > 0.05).

### RCAN 1-4 protein content before and after a single session of HIIE

**Figure 3:**
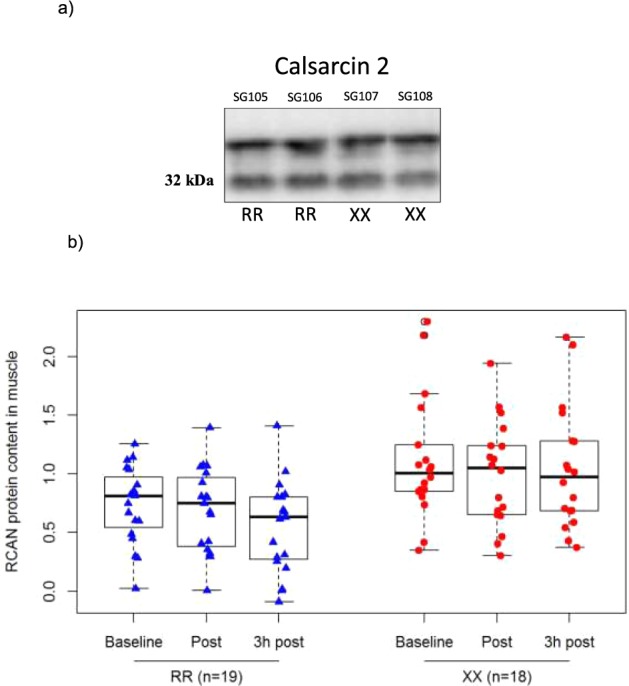
(**a**) Representative images of calsarcin-2 protein content in ACTN3 RR vs. ACTN3 XX participants at baseline. Calsarcin-2 protein content did not differ between muscle samples obtained from ACTN3 XX and ACTN3 RR humans (**b**) A main effect of genotype for RCAN 1-4 protein content was demonstrated, but there was no significant interaction effect. Box blot displaying RCAN1-4 protein content in ACTN3 RR vs. XX participants. RCAN 1-4 protein content is in arbitrary units (a.u.). The centre rectangle includes the mean and the interquartile range (IQR). The “whiskers” above and below the box show the locations of the maximum and minimum (excluding outliers – defined as ≥0.15 × IQR above the third quartile or below the first quartile and indicated by an unfilled circle).

Resting calsarcin-2 content was similar between *ACTN3* RR and *ACTN3* XX (Fig. 3a). There was a main effect of genotype for RCAN 1-4 protein content (P = 0.004), with greater RCAN1-4 protein content in the muscle of *ACTN3* 577XX versus *ACTN3* 577RR humans (1.3 fold; P = 0.004). Despite the main effect of genotype for RCAN1-4 content, there was no significant interaction between genotype and the time at which the baseline biopsy was taken relative to the HIIE session (p = 0.12) (Fig. 3b).

### The influence of α-actinin-3 deficiency on mitochondrial-related gene expression

**Figure 4:**
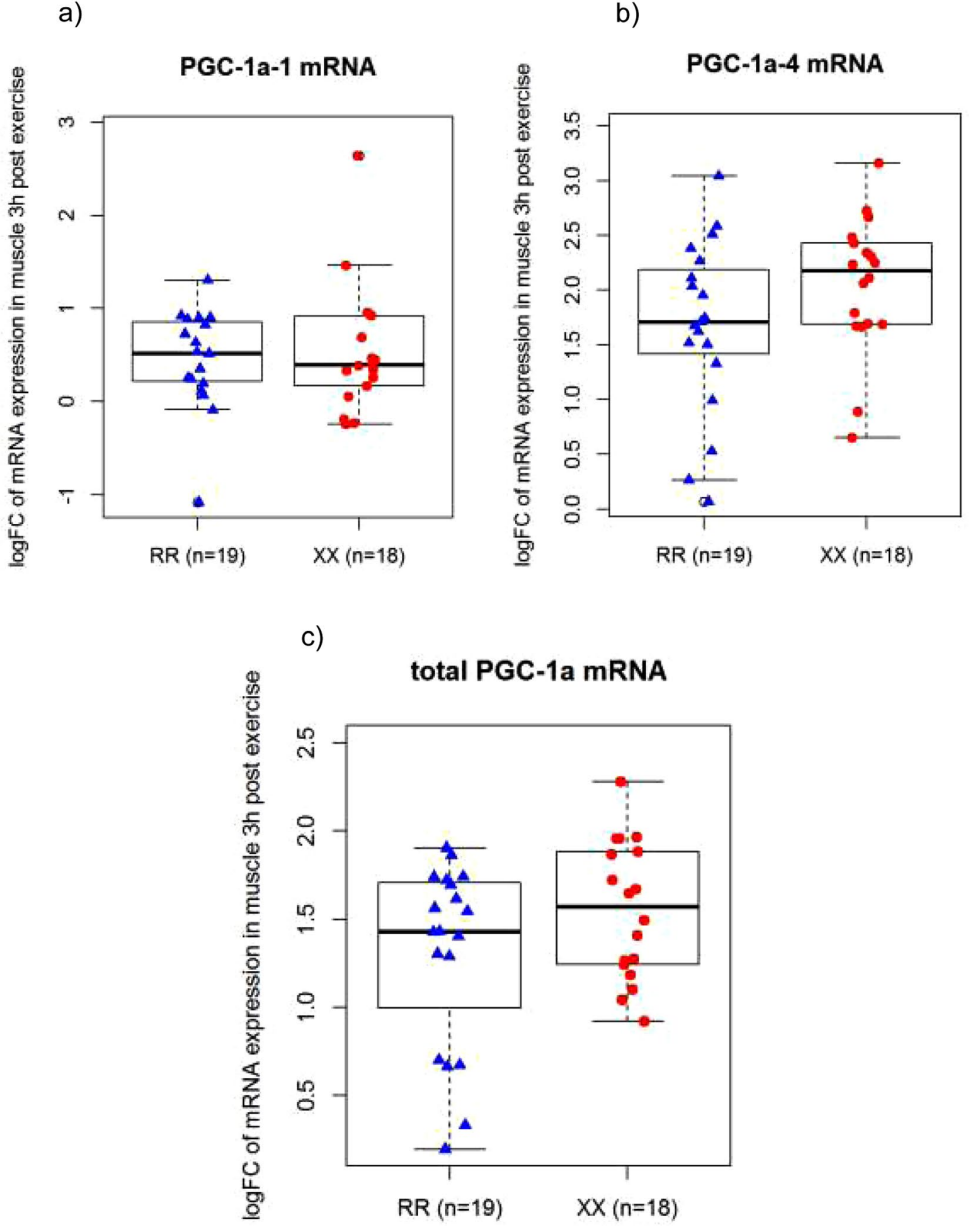
Log fold change from baseline to 3 h post exercise for RR and XX participants for: (**a**) PGC-1α-1 mRNA expression. P-values: for exercise < 0.05, for genotype = 0.39, and for interaction (exercise × genotype) = 0.58 (**b**) PGC-1α-4 mRNA expression. P-values: for exercise < 0.05, for genotype = 0.25, and for interaction (exercise × genotype) = 0.13 and (**c**) total PGC-1α mRNA expression. P-values: for exercise <0.05, for genotype = 0.76, for the interaction (exercise × genotype) = 0.36. Box blot displaying PGC1α-1 mRNA values in ACTN3 RR vs. XX participants. The centre rectangle includes the mean and the interquartile range (IQR). The “whiskers” above and below the box show the locations of the maximum and minimum (excluding outliers – defined as ≥0.15 × IQR above the third quartile or below the first quartile and indicated by an unfilled circle).

The results for PGC-1α total, PGC-1α-1, and PGC-1α-4 gene expression are illustrated in Fig. 4. Although there was a main effect of exercise for all three PGC-1α gene transcripts, there was no main effect of genotype and no interaction effect. Gene expression analyses of COX-1, RCAN 1-4, HSP70, VEGF, PDK4, SOD-1, MFN2, SDHB and NUDFB3 immediately post and 3 h post exercise are shown in Table 2. Again, there was no evidence for any difference between *ACTN3* XX and *ACTN3* RR genotypes, although there were significant time effects (p < 0.05) for some of the tested genes (COX-1, RCAN 1-4, HSP70, VEGF and PDK4) (Table 3).

**Table 3:** Gene expression in arbitrary units (a.u.) for COX4-1, MFN2 C, RCAN1-4, SDHB, SOD-1, PDK4, HSP70, VEGF and NUDFB3.

Gene	ACTN3 Genotype	Pre Exercise	Post Exercise	+3 h Exercise
*MFN2 C*	RR n = 19	3.57 (1.63)	3.21 (1.70)	3.65 (1.78)
	XX n = 18	3.66 (1.28)	3.32 (1.57)	4.22 (1.95)
*COX4-1*	RR n = 14	5.11 (3.12)	4.44 (2.82)	4.32 (2.54)
	XX n = 13	4.59 (2.04)	4.14 (2.68)	5.08 (2.51)
RCAN1-4*	RR n = 14	0.72 (0.42)	0.69 (0.31)	1.20 (0.96)
	XX n = 13	1.06 (0.96)	0.96 (0.73)	1.32 (2.07)
*SDHB*	RR n = 14	1.04 (0.56)	0.84 (0.26)	0.98 (0.46)
	XX n = 13	0.74 (0.54)	0.73 (0.53)	0.78 (0.38)
*SOD-1*	RR n = 14	0.97 (0.50)	0.90 (0.23)	0.92 (0.29)
	XX n = 13	0.67 (0.40)	0.69 (0.31)	0.69 (0.25)
PDK4*	RR n = 14	1.59 (1.21)	2.25 (1.57)	9.63 (5.50)
	XX n = 13	2.18 (1.61)	4.00 (3.81)	9.79 (6.53)
HSP70*	RR n = 14	1.27 (0.53)	2.56 (0.99)	3.55 (2.22)
	XX n = 13	1.02 (0.42)	1.99 (1.26)	2.95 (1.68)
VEGF*	RR n = 14	0.64 (0.20)	0.57 (0.23)	1.19 (0.45)
	XX n = 13	0.46 (0.19)	0.64 (0.64)	0.96 (0.30)
*NUDFB3*	RR n = 14	1.48 (0.53)	1.91 (0.58)	1.71 (0.55)
	XX n = 13	1.15 (0.66)	1.42 (0.60)	1.39 (0.68)

Values in parentheses represent the mean ± SEM. There were no significant genotype effects for any of these genes (p < 0.05). *Significant time effect (p < 0.05).

### PGC-1α Protein expression in *ACTN3* RR and XX humans

**Figure 5:**
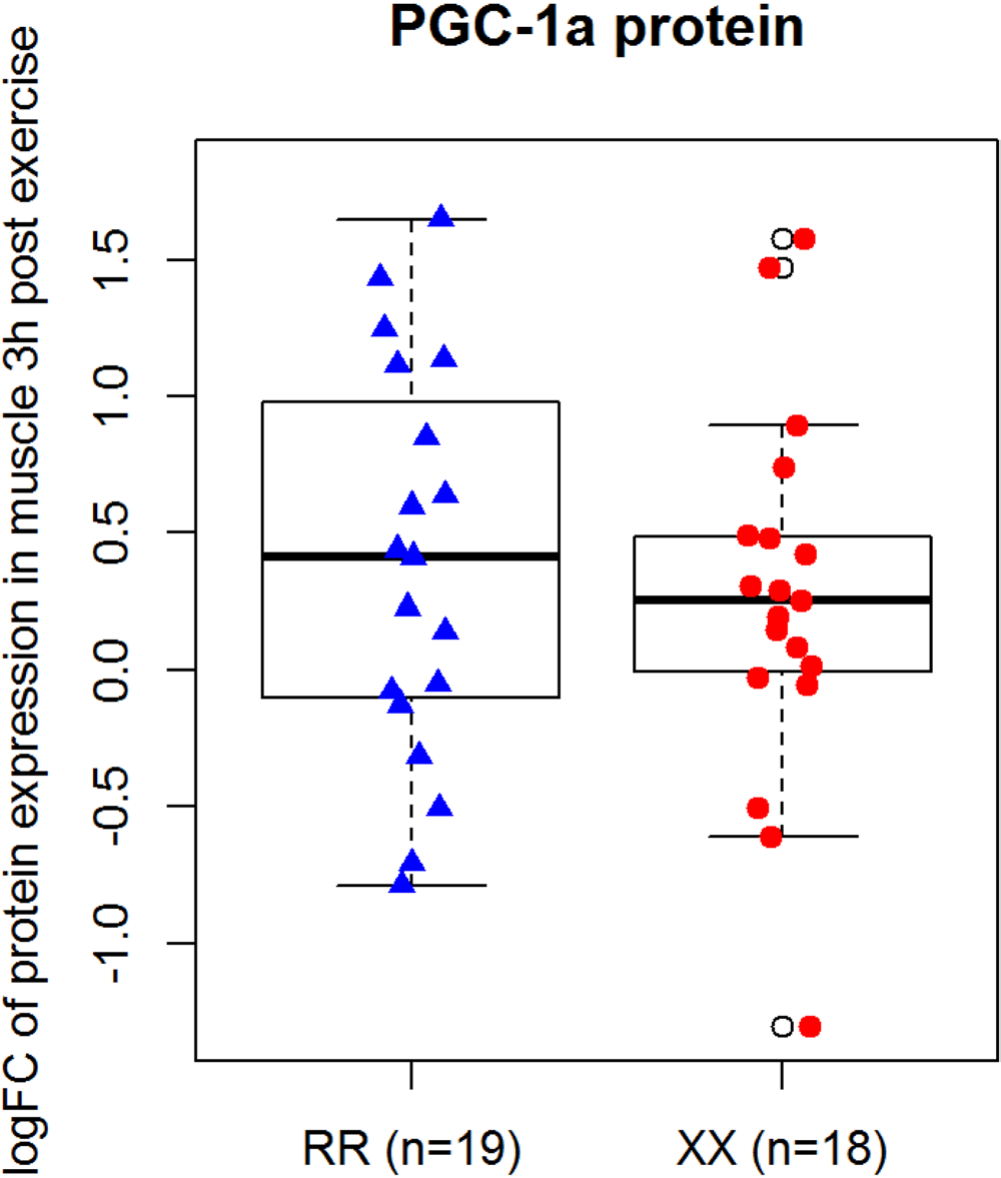
Log fold change from baseline to 3 h post exercise for PGC 1-a protein content in the muscle of RR and XX partitipants (3 h post exercise/pre exercise). Box blot displaying PGC1α-1 relative protein abundance in ACTN3 RR vs. XX participants. The centre rectangle includes the mean and the interquartile range (IQR). The “whiskers” above and below the box show the locations of the maximum and minimum (excluding outliers – defined as ≥0.15 × IQR above the third quartile or below the first quartile and indicated by an unfilled circle).

PGC-1α total protein was significantly increased after HIIE (p-value for exercise < 0.001). However, there was no significant main effect of genotype (p = 0.49), and no significant interaction effect (p = 0.77) (Fig. 5).

### Training-induced changes in endurance-related phenotypes

**Figure 6:**
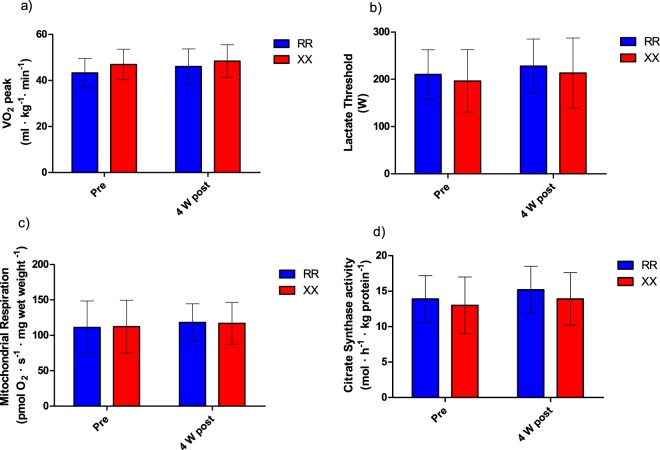
Mean ± SD for (**a**) VO_2peak_, (**b**) Lactate Threshold (**c**) Mitochondrial Respiration (**d**) Citrate Synthase activity pre and post 4 weeks high intensity interval training (HIIT) in ACTN3 RR and ACTN3 XX humans.

Consistent with our pre-training measurements, there were no main effects of training or significant differences for the LT, VO_2peak_, W_peak_, or time-trial performance (Table 4) between *ACTN3 XX* and *ACTN3 RR* participants after 4 weeks of completing the same high intensity interval training (Fig. 6).

**Table 4:** Endurance-related phenotypes measured after 4 weeks of training: Lactate threshold, VO_2 peak_, W_peak_, Time-trial performance, mitochondrial respiration, and citrate synthase activity for each of the two ACTN3 genotypes.

Phenotype	Genotype	
	RR (n = 19)	XX (n = 18)
Lactate Threshold (W)	227.9 (57.2)	213.4 (74.0)
VO_2 peak_ (mL•min^−1^•kg^−1^)	46.1 (7.6)	48.4 (7.1)
Lactate Threshold as % of Power Peak (%)	73.1 (5.6)	72.7 (7.3)
Power Peak (W_peak_)	309.2 (63.1)	288.6 (76.4)
Time-Trial Performance (s)	2167 (242)	2242 (231)
Mitochondrial Respiration (pmol O_2_/s^−1^ ·mg wet weight^−1^)	118.2 (26.4)	116.9 (29.7)
Citrate synthase activity (mol·h^−1^ · kg protein^−1^)	15.2 (3.3)	13.9 (3.7)

Values are reported as Mean (SD). No significant difference was demonstrated for any of these phenotypes between RR and XX genotypes (p > 0.05).

## Discussion

The high percentage of the population who are deficient for the α-actinin-3 protein has allowed us to employ a unique “human knockout” model to investigate the influence of α-actinin-3 protein on baseline endurance characteristics, as well as the response to both a single exercise session and four weeks of exercise training. Following the initial screening, we included 19 *ACTN3 RR* and 18 *ACTN3* XX participants into the main study. In addition to genotyping in duplicate, we confirmed that the α-actinin-3 protein was present in *ACTN3* RR and not *ACTN3* XX participants. At baseline, absence of the α-actinin-3 protein was accompanied by a compensatory increase in α-actinin-2 protein content in *ACTN3* XX vs *ACTN3* RR humans. There was, however, no evidence for significant differences in physiological characteristics, or endurance-related phenotypes and mitochondrial characteristics, between *ACTN3* XX and *ACTN3 RR* participants. While there was a main effect of genotype for RCAN1-4 protein content, there was no significant interaction effect for exercise-induced changes in any of the measured genes or proteins associated with mitochondrial biogenesis. Consistent with our results for exercise-induced changes in mRNA, absence of α-actinin-3 protein has no influence on the response to training for any of the physiological or mitochondrial characteristics accessed. Thus, in contrast to studies in mice, absence of *ACTN3* protein in humans does not influence the response to a single session of endurance exercise or exercise training.

### The influence of the ACTN3 gene on physiological parameters at baseline

There were no significant differences in the lactate threshold, VO_2peak_, W_Peak_, cycling time-trial performance, CS activity, or maximal ADP-stimulated mitochondrial respiration, between *ACTN3* XX and *ACTN3* RR humans (Table 2). These variables were chosen as both VO_2peak_ and the lactate threshold have been reported to be important determinants of endurance performance^35,41^. Mitochondrial respiration and CS activity have also been associated with endurance performance in humans^24^. However, our results indicate there is no influence of the *ACTN3* polymorphism on any of these key endurance phenotypes at baseline. When we repeated the assessments after four weeks of high-intensity interval training, we also observed no influence of the *ACTN3* polymorphism on these endurance-related phenotypes. These findings are in line with the results from a large case-control study^12^, cross-sectional studies^42–45^ recent meta-analyses^46–48^ and our quantitative analysis^13^ that also found no evidence of an association between the *ACTN3* polymorphism and endurance-related phenotypes in humans.

### The influence of ACTN3 gene on the protein content of calsarcin-2 & RCAN1-4

It has been suggested that the molecular basis for the effects of α-actinin-3 deficiency on muscle properties may be related to the activity of calcineurin, which indirectly associates with sarcomeric α-actinins at the Z-disk^14^. It is well established that calcineurin signalling induces activation of the slow myogenic program and a shift in muscle fibre attributes toward a slower, more oxidative phenotype^14^. RCAN1-4 is a downstream target of calcineurin, which is increased upon activation by calcineurin and provides a sensitive, indirect measure of calcineurin activity^18,49^. Consistent with the results in mice^14^, there was a main effect of genotype for RCAN1-4 protein content (*ACTN3* XX > *ACTN3* RR; P = 0.004). To assess whether the elevated RCAN 1-4 content in *ACTN3* XX humans could be attributed to differential expression of calsarcin-2 (which inhibits calcineurin activity^50^), the content of calsarcin-2 expression was also measured. Consistent with observations in mice^14^, the expression of calsarcin-2 did not differ between muscle samples obtained from *ACTN3* XX and *ACTN3* RR humans and therefore does not account for the increased RCAN1-4 protein content associated with α-actinin-3 deficiency.

In mice, there was no significant difference for the levels of RCAN1-4 protein in KO vs WT animals (1.3 fold difference; P = 0.057), but a 2.9-fold greater RCAN1-4 protein content was observed in KO muscle compared with WT muscles 3 days after completing an endurance exercise (p = 0.004)^14^. Consistent with their after-exercise RCAN1-4 results^14^, a 1.9-fold greater calcineurin activity in exercised KO compared with WT muscles was also observed, although this difference did not reach statistical significance. Given that our resting muscle biopsies were performed 48 to 72 h after prior endurance exercise (a 20-k TT lasting ~ 40 minutes), our results can best be compared to the after-exercise results reported by Seto *et al*.^14^. In this regard, we also observed a greater RCAN1-4 protein content in the muscle of *ACTN3* 577XX versus *ACTN3* 577RR humans two to three days after exercise (1.3 fold; P = 0.004). Despite the main effect of genotype for RCAN1-4 content, there was no significant interaction between genotype and time (i.e., whether our biopsies were taken before or after a session of high-intensity interval exercise).

One factor that may have contributed to some of the differences observed between mice and humans is that murine muscle contains a significantly higher percentage of fast-twitch fibres compared with human muscle^51^, and the ACTN3 gene is exclusively expressed in fast-twitch skeletal muscles^52^. The muscles analysed in the study by Seto *et al*.^14^ contained less than 1% type I fibres, and therefore any effects of α-actinin-3 protein would have been enhanced in their mouse model. In line with this hypothesis, exhaustive exercise in rats increased RCAN1-4 protein levels in the gastrocnemius, but not the soleus (the gastrocnemius contains a higher percentage of fast-twitch fibres compared to soleus)^53^. Recent studies with single muscle fibres have also observed that the greater maximal unloading velocity in the muscle of *ACTN3* RR compared to *ACTN3* XX genotypes likely contributes to enhanced whole-muscle performance during high-velocity contractions^54^. Future studies in single muscle fibres may shed more light on the influence of the *ACTN3* gene on human performance and exercise-induced cell signalling. It is also worth noting that only men were recruited in the present study, while the previous human study only recruited females^14^. It has previously been reported that the effect of *ACTN3* genotype on endurance performance is more pronounced in female compared with male athletes^3^. Furthermore, the human *ACTN3* XX participants in the study of Seto *et al*.^14^, that showed increased RCAN1-4 expression compared with *ACTN3* RR participants (P = 0.004) were quite old (mean age 49 years, with a very large age range (33–77 years) and the sample size was small (n = 5). Future research should examine if the effects of *ACTN3* genotype are enhanced in both females and aged muscle.

### The influence of ACTN3 R577X polymorphism on mitochondrial-related gene expression in response to exercise

Based on research with mice reporting a significant effect of *ACTN3* genotype on training-induced changes in endurance performance, it was hypothesised that *ACTN3* genotype would affect the exercise-induced increase in nuclear genes encoding mitochondrial proteins. However, despite a significant exercise-induced increase of all the PGC-1α gene transcripts and PGC-1α protein levels in both genotypes (p < 0.05), there was no significant main effect for genotype or an interaction effect. Similarly, there was no significant main effect for genotype or an interaction effect for the exercise-induced increase of other nuclear genes encoding mitochondrial proteins. These finding are consistent with the absence of a significant difference between *ACTN3* XX and *ACTN3* RR individuals for the exercise-induced elevation of RCAN 1-4 protein content (i.e., immediately post and 3 hours post exercise versus baseline).

Exercise-induced changes in some mitochondria-related genes have been reported to be intensity-dependent^24^ with greater increases in PGC-1α a mRNA observed after exercise performed at 100% VO_2max_ compared with 73% VO_2max_ (7.9 vs 4.3 fold^55^). Based on these previous observations, we chose to investigate the effects of *ACTN3* genotype on changes in mitochondria-related genes in response to a single session of high-intensity interval exercise. It is worth noting, however, that calcineurin has been reported to contribute to changes in muscle fibre phenotype in response to low-amplitude, sustained calcineurin activity^56^. Therefore, future research should examine whether the effects of *ACTN3* genotype on exercise-induced changes in calcineurin-related signalling, and gene expression, are more pronounced in response to other forms of exercise (e.g., lower-intensity continuous exercise).

### ACTN3 genotype and training response

Consistent with the gene-expression results, there was no effect of genotype on changes in the LT, VO_2peak_, W_Peak_, or 20-k TT performance, after four weeks of HIIT. While these results contrast with post-training observations after four weeks of continuous exercise training in *actn3* KO mice^14^, they are consistent with research that has not identified a relationship between the *ACTN3* polymorphism and endurance-related phenotypes in humans^12,13,42–48^. We chose to investigate the effects of *ACTN3* genotype on physiological adaptations to high-intensity interval training, as this type of training has been reported to be equally or more effective than continuous exercise training for improving aerobic fitness (VO_2max_)^57^ and promoting mitochondrial adaptations in muscle^58^. However, future research should examine whether the effects of *ACTN3* genotype on training-induced changes in physiological parameters differ in response to other forms of exercise (e.g., continuous, moderate-intensity exercise) or longer (>4 weeks) exercise-training periods.

## Conclusions

In conclusion, the results of the present study showed a compensatory increase in *α-actinin-2* protein content at baseline, with no evidence for a greater adaptive response to a single session of high-intensity interval exercise in participants with the *ACTN3* XX genotype. This analysis is the first well-powered study in humans to investigate potential molecular pathways that might contribute to the hypothesised influence of *ACTN3* R577X genotype on the response to endurance exercise. These results, combined with the absence of significant differences in baseline endurance characteristics, as well as the response to four weeks of exercise training, add to the growing body of literature suggesting *ACTN3* genotype does not have a major influence on endurance performance or the response to endurance training in humans. Despite the absence of significant differences, the use of naturally occurring “human knockouts” could still be a useful methodological approach to investigate the role of the loss of function of other proteins on the response to exercise in humans.

## Supplementary information

Representative Blots

Raw Data
