# Supplementary material for: A “human knockout” model to investigate the influence of the α-actinin-3 protein on exercise-induced mitochondrial adaptations
Source: Sci Rep. 2019 Sep 3;9:12688. doi: 10.1038/s41598-019-49042-y (PMC6722100; doi:10.1038/s41598-019-49042-y)
Supplement: Supplementary file 1 — Representative Blots [file 41598_2019_49042_MOESM1_ESM.pdf]

Supplementary Information

(cropped Blots & full length blots)

A human knockout model to investigate the influence of the α-actinin-3 protein on exercise-induced mitochondrial adaptations

I.D. Papadimitriou[1], N. Eynon[1,2], X. Yan[1], F. Munson[1], M. Jacques[1], J. Kuang[1], S. Voisin[1], K.N. North [2], D.J. Bishop[1,3]

[1] Institute for Health and Sport (iHeS), Victoria University, Melbourne, Australia

[2] Murdoch Children's Research Institute, Melbourne, Australia

[3] School of Medical & Health Sciences, Edith Cowan University, Joondalup, Australia

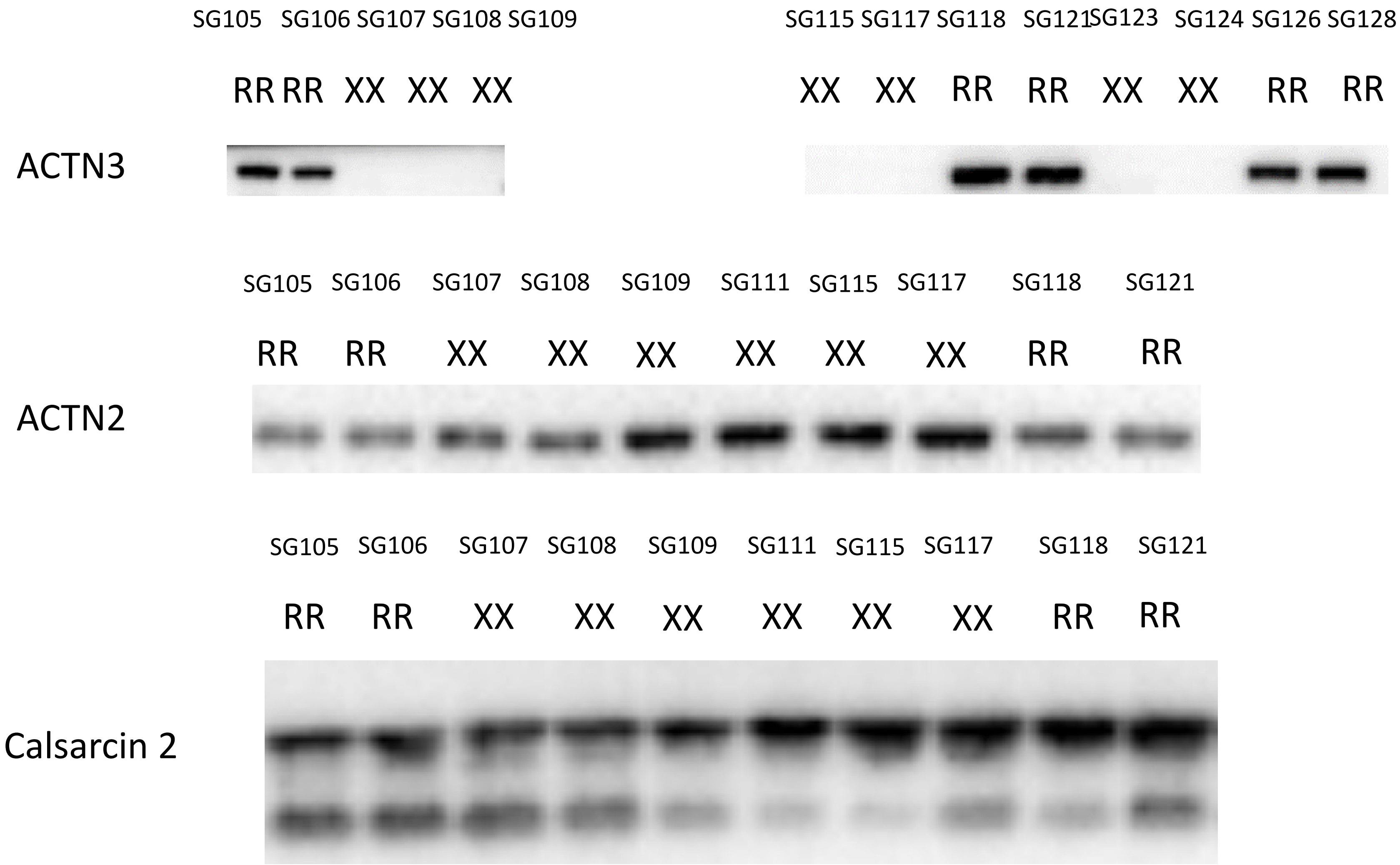
SG105 SG106 SG107 SG108 SG109
SG115 SG117 SG118 SG121 SG123 SG124 SG126 SG128
RR RR XX XX XX
XX XX RR RR XX XX RR RR
ACTN3
SG105 SG106 SG107 SG108 SG109 SG111 SG115 SG117 SG118 SG121
RR RR XX XX XX XX XX XX RR RR
ACTN2
SG105 SG106 SG107 SG108 SG109 SG111 SG115 SG117 SG118 SG121
RR RR XX XX XX XX XX XX RR RR
Calsarcin 2

ACTN3 Blots

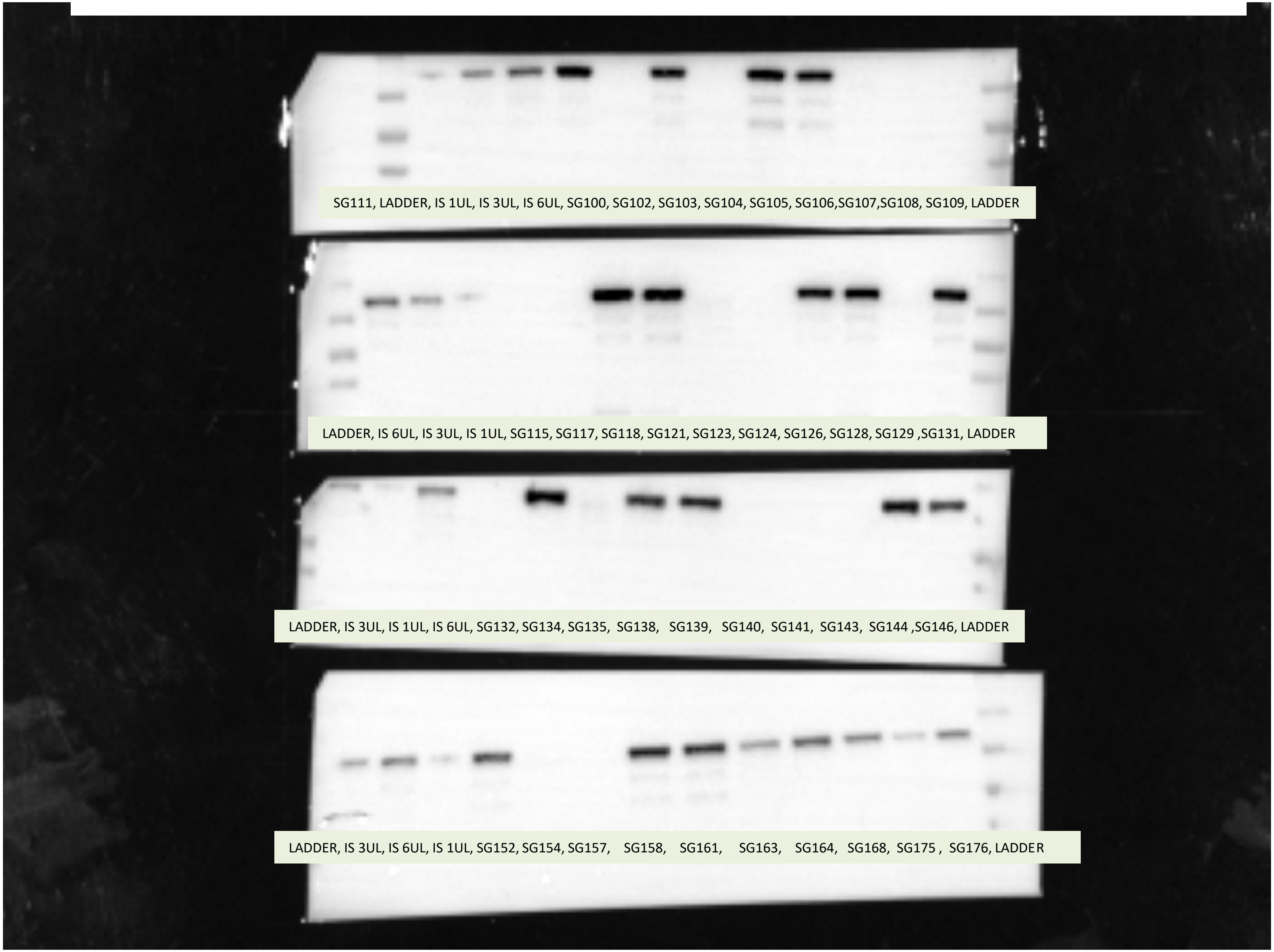
SG111, LADDER, IS 1UL, IS 3UL, IS 6UL, SG100, SG102, SG103, SG104, SG105, SG106,SG107,SG108, SG109, LADDER
LADDER, IS 6UL, IS 3UL, IS 1UL, SG115, SG117, SG118, SG121, SG123, SG124, SG126, SG128, SG129 ,SG131, LADDER
LADDER, IS 3UL, IS 1UL, IS 6UL, SG132, SG134, SG135, SG138, SG139, SG140, SG141, SG143, SG144 ,SG146, LADDER
LADDER, IS 3UL, IS 6UL, IS 1UL, SG152, SG154, SG157, SG158, SG161, SG163, SG164, SG168, SG175 , SG176, LADDER

ACTN2 BLOTS

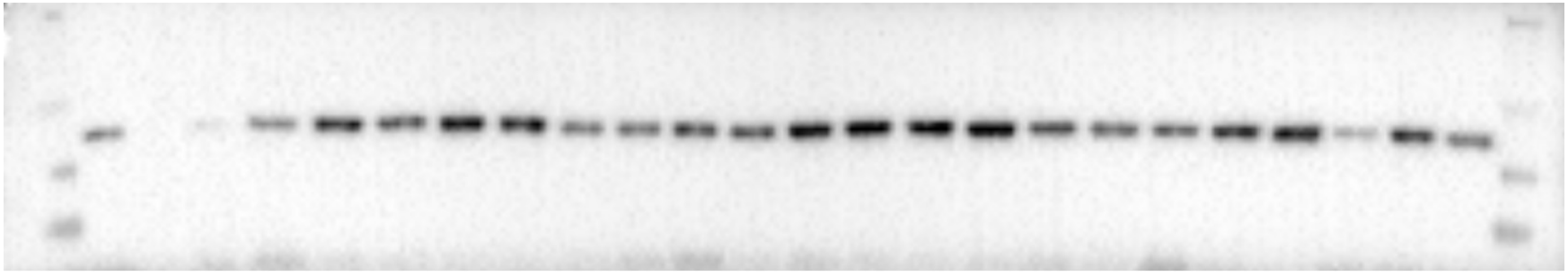

LADDER, IS 6ul, IS 1ul, IS 3ul, IS 9ul, SG100, SG102, SG103, SG104, SG105, SG106, SG107, SG108, SG109, SG111, SG115, SG117, SG118, SG121, SG123, SG124, SG126, SG128, SG129, SG131, LADDER

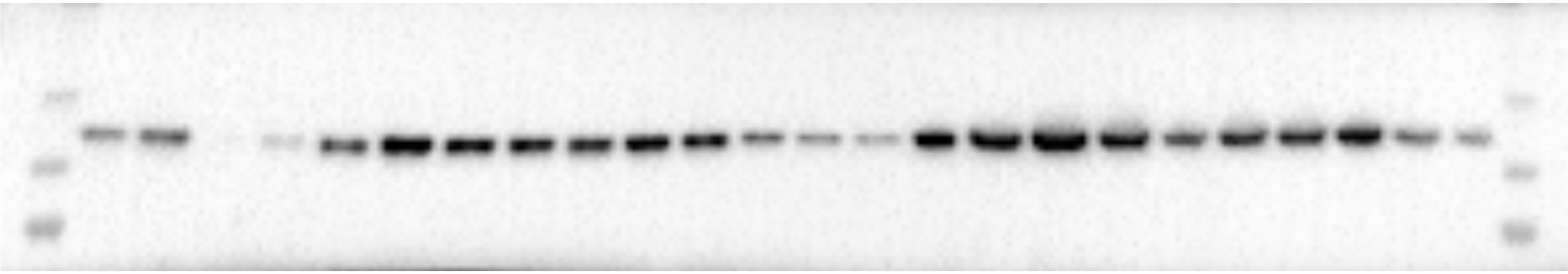

LADDER, IS 6ul, IS 9ul, IS 1ul, IS 3ul, SG132, SG134, SG135, SG138, SG139, SG140, SG141, SG143, SG144, SG146, SG152, SG154, SG157, SG158, SG161, SG163, SG164, SG168, SG175, SG176, LADDER

CALSARCIN-2 BLOTS

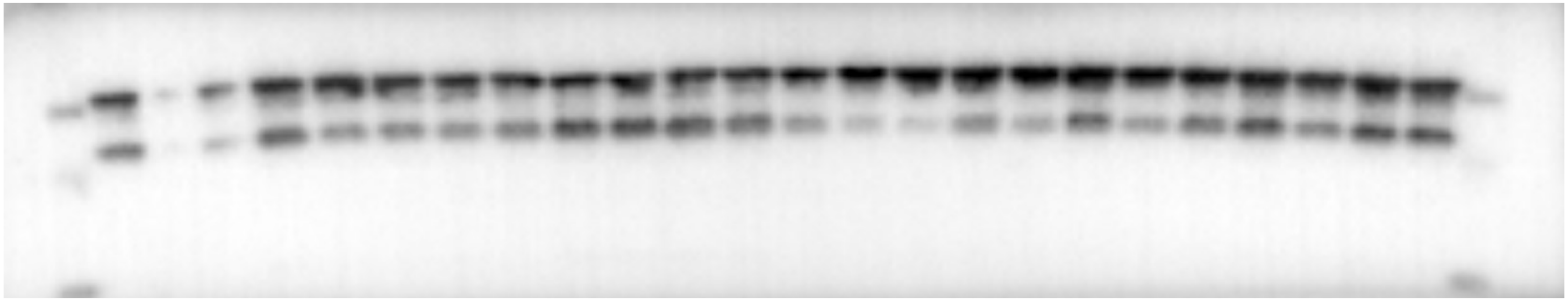

LADDER, IS 6ul, IS 1ul, IS 3ul, IS 9ul, SG100, SG102, SG103, SG104, SG105, SG106, SG107, SG108, SG109, SG111, SG115, SG117, SG118, SG121, SG123, SG124, SG126, SG128, SG129, SG131, LADDER

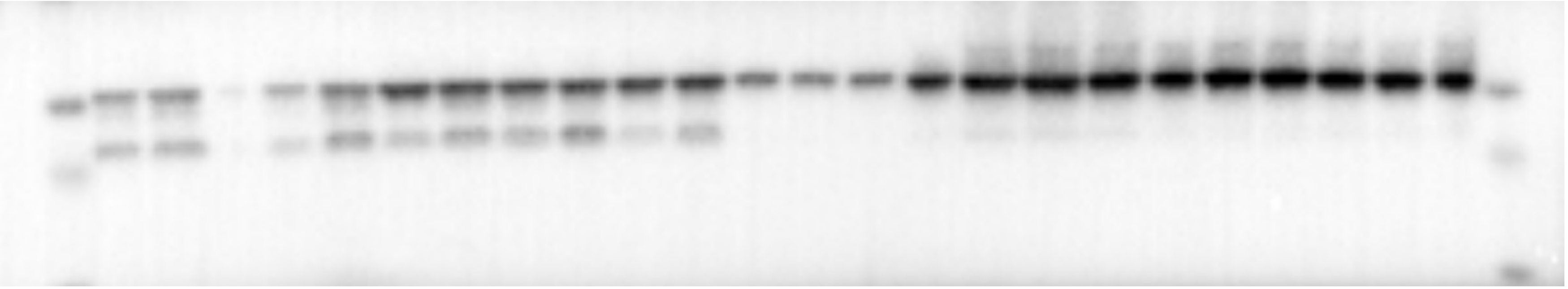

LADDER, IS 6ul, IS 9ul, IS 1ul, IS 3ul, SG132, SG134, SG135, SG138, SG139, SG140, SG141, SG143, SG144, SG146, SG152, SG154, SG157, SG158, SG161, SG163, SG164, SG168, SG175, SG176, LADDER
